# Value of diffusion-weighted imaging for monitoring tissue change during magnetic resonance-guided high-intensity focused ultrasound therapy in bone applications: an *ex-vivo* study

**DOI:** 10.1186/s41747-018-0041-x

**Published:** 2018-05-10

**Authors:** Sharon L. Giles, Jessica M. Winfield, David J. Collins, Ian Rivens, John Civale, Gail R. ter Haar, Nandita M. deSouza

**Affiliations:** 1 MRI Unit, The Royal Marsden NHS Foundation Trust, London, UK; 2 Cancer Research UK Cancer Imaging Centre, Division of Imaging and Radiotherapy, The Institute of Cancer Research, London, UK; 3 Therapeutic Ultrasound, Division of Imaging and Radiotherapy, The Institute of Cancer Research, London, UK

**Keywords:** Bone metastases, Diffusion-weighted imaging (DWI), Magnetic resonance imaging (MRI), Periosteum, Thermal ablation

## Abstract

**Background:**

Magnetic resonance (MR)-guided high-intensity focused ultrasound (HIFU) can palliate metastatic bone pain by periosteal neurolysis. We investigated the value of diffusion-weighted imaging (DWI) for monitoring soft tissue changes adjacent to bone during MR-guided HIFU. We evaluated the repeatability of the apparent diffusion coefficient (ADC) measurement, the temporal evolution of ADC change after sonication, and its relationship with thermal parameters.

**Methods:**

*Ex-vivo* experiments in lamb legs (*n* = 8) were performed on a Sonalleve MR-guided HIFU system. Baseline proton resonance frequency shift (PRFS) thermometry evaluated the accuracy of temperature measurements and tissue cooling times after exposure. PRFS acquired during sonication (*n* = 27) was used to estimate thermal dose volume and temperature. After repeat baseline measurements, DWI was assessed longitudinally and relative ADC changes were derived for heated regions.

**Results:**

Baseline PRFS was accurate to 1 °C and showed that tissues regained baseline temperatures within 5 min. Before sonication, coefficient of variation for repeat ADC measurements was 0.8%. After sonication, ADC increased in the muscle adjacent to the exposed periosteum, it was maximal 1–5 min after sonication, and it significantly differed between samples with persistent versus non-persistent ADC changes beyond 20 min. ADC increases at 20 min were stable for 2 h and correlated significantly with thermal parameters (ADC versus applied acoustic energy at 16–20 min: *r* = 0.77, *p* < 0.001). A 20% ADC increase resulted in clear macroscopic tissue damage.

**Conclusions:**

Our preliminary results suggest that DWI can detect intra-procedural changes in ex-vivo muscle overlying the periosteum. This could be useful for studying the safety and efficacy of clinical MR-guided HIFU bone treatments.

## Keypoints

- DWI detected intra-procedural changes in muscle overlying the periosteum during MR-guided HIFU to bone
- ADC measurements in these regions were highly repeatable
- The magnitude of early ADC change was indicative of sustained changes later
- ADC changes correlated with both applied and measured thermal parameters
- Intra-procedural DWI is potentially informative about safety and efficacy of treatments

## Background

Magnetic resonance (MR)-guided high-intensity focused ultrasound (HIFU) offers a safe and effective treatment option for the palliation of pain from bone metastases by thermal neurolysis at the periosteum [1, 2]. To indicate whether ablative tissue temperatures are likely to have been reached at the periosteum during treatments (> 55 °C for > 1 s) [3], thermal monitoring is undertaken using the proton resonance frequency shift (PRFS) method. However, because PRFS is ineffective in bone [4] and is unable to resolve the periosteal nerves, thermal neurolysis can only be inferred by monitoring heating in adjacent, near-field muscle regions overlying the bone surface. Conventionally, gadolinium contrast-enhanced T1-weighted imaging is used to visualise the extent of any induced tissue ablation after treatment [5] but it cannot be used during treatments as it may influence the accuracy of PRFS thermometry [6] and confound post-treatment assessment [7]. Unfortunately, unenhanced T1- and T2-weighted imaging are less sensitive than contrast-enhanced imaging for detection of thermal ablation around bone [8].

Diffusion-weighted imaging (DWI) is an alternative contrast mechanism that has been shown to identify tissue changes during pre-clinical thermal treatments of canine prostate tumours [9, 10] and murine tumour models [11]. Quantification of DWI changes using the apparent diffusion coefficient (ADC) has also been used as part of a multi-parametric MR assessment of residual disease after clinical prostate HIFU treatments [12]. In addition, numerous studies have evaluated DWI and ADC for indicating response after clinical treatments of uterine fibroids using HIFU or embolisation techniques [13–17]. However, only one study has evaluated DWI for assessing response after palliative MR-guided HIFU treatments of bone metastases [18]. Furthermore, DWI and ADC have not been used for monitoring intra-procedural tissue changes during palliative bone treatments and any relationship between DWI changes and thermal parameters in this setting has not been explored.

We therefore sought to investigate the principle of using DWI to monitor tissue changes during MR-guided HIFU therapy in bone applications by: 1) evaluating the repeatability of ADC measurement in muscle regions overlying the periosteum; 2) investigating the temporal evolution of ADC change in these regions after exposure; 3) evaluating the relationship between prescribed and PRFS-estimated thermal parameters in these regions; 4) assessing potential correlations between ADC changes and PRFS-derived temperature and thermal dose estimates; and 5) validating imaging findings with macroscopic tissue changes after exposure.

## Methods

### Model system

**Fig. 1:**
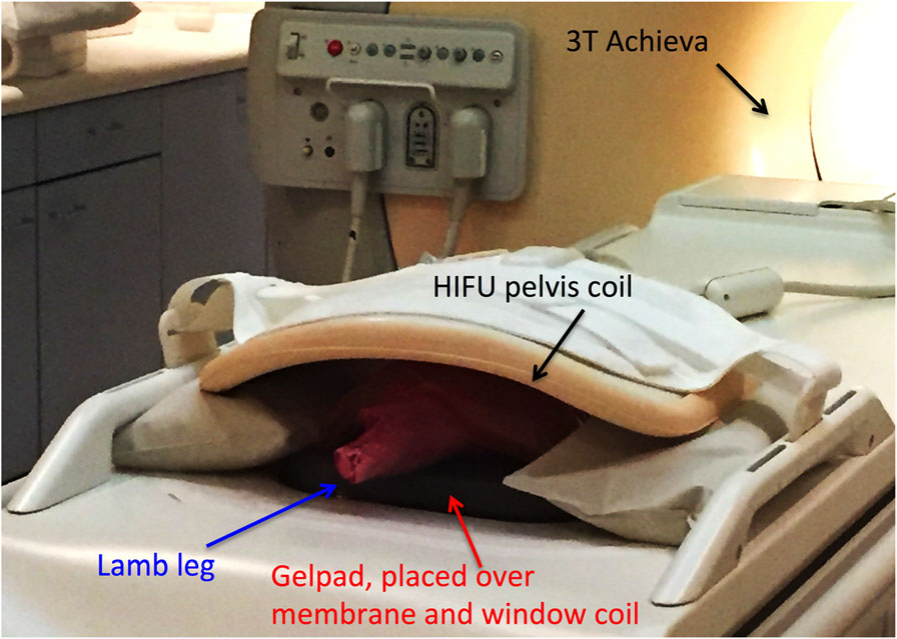
A lamb leg sample shown mounted for sonication in close acoustic contact with a dampened Aquaflex gelpad. The gelpad was acoustically coupled to the membrane covering the high-intensity focused ultrasound (HIFU) window coil using de-gassed water. The mobile, 256-element, 14 cm radius-of-curvature, phased-array ultrasound equipment is located beneath this membrane. The lamb leg sample was secured in place using the HIFU pelvis coil. The pelvis and window coil were operated in dual coil mode for imaging

Experiments were performed in *ex-vivo* lamb legs (*n* = 8) obtained from a butcher. These were available at a uniform weight of approximately 2 kg, with the skin and subcutaneous fat mostly removed. A 3T Achieva MR/Sonalleve HIFU system (Philips Healthcare, Best, The Netherlands/Vantaa, Finland) was used. Experiments were performed at room temperature (19–22 °C) rather than a physiological temperature. Lamb leg samples were removed from refrigeration at least 12 h before experiments and placed in the scan room to equilibrate to room temperature for at least 4 h. Prior to ultrasound exposure, MR-SPOT markers (Beekley Medical, Bristol, CT, USA) were inserted into the uppermost surface of the lamb legs to act as spatial reference markers. Samples were then positioned in direct acoustic contact with a de-gassed water-dampened Aquaflex gelpad (Parker Laboratories Inc., Fairfield, NJ, USA) placed over the acoustically transparent membrane that covers the Sonalleve transducer (Fig. 1).

### Planning of sonications

Prior to exposures, limited coverage three-dimensional gradient-echo T1-weighted images were acquired at interfaces between the membrane, gelpad, and lamb leg samples. This was to verify the exclusion of trapped air which would have adversely affected HIFU transmission and reduced targeting accuracy. High-resolution (1 × 1 × 1 mm^3^ voxel) three-dimensional gradient-echo T1-weighted imaging of each whole sample was then acquired and exported to the Sonalleve for treatment planning. Sonications were planned with the focus located on the outer bony cortex (the direct approach [19]). In each sample, the focus was placed 3–4 cm deeper than the proximal tissue surface in contact with the gelpad. Care was taken to ensure that sonications were placed far enough apart to avoid heating the location of neighbouring exposures and were planned to be in line with the MR markers. Sonications were delivered using treatment “cells” of a particular diameter. The smallest cell (2 mm diameter) was obtained from a sonication in a single position, whilst larger cells (4, 8, or 12 mm diameter) were obtained by electronically steering the focal point in predefined circular trajectories around a central point [20].

### Baseline temperature measurements

As ADC is temperature dependent [21], it was important to understand the temperature behaviour of the ex-vivo lamb leg samples before and after sonication. Room temperature was measured using a standard alcohol thermometer. All other temperature measurements were made from a PRFS thermometry sequence using an echo planar imaging (EPI) accelerated, multi-slice two-dimensional gradient-echo T1-weighted sequence, acquired as a dynamic series (repetition time 25 ms, echo time 16 ms, flip angle 18^o^, EPI factor 11, fat suppression *ProSet*, number of signal averages 2, voxel size 2.1 × 2.1 × 7.0 mm^3^, field of view 400 × 300 mm^2^, dynamic scan time 3 s). Data were obtained from three 7 mm thick slices automatically placed orthogonally through the treatment focus. In lamb leg number 1, six separate 1 min acquisitions of PRFS data (23 dynamics) were first performed in each of three separate cortical locations to determine the stability and accuracy of the PRFS predicted temperature. This was compared to the measured room/lamb leg temperature. The same sequence was then acquired before, during, and after sonication of a 4 and 12 mm cell at the maximum power permissible at each cell size (190 W for the 4 mm cell, 80 W for the 12 mm cell). Data were obtained for the maximum cooling period for which temperature data were available.

### Imaging experiments

DWI experiments were undertaken in lamb legs from number 2 to number 7. To obtain baseline repeatability estimates, a single-shot EPI DWI sequence with spectrally attenuated inversion recovery and gradient reversal off-resonance fat suppression was acquired twice in each sample before sonications, using b-values of 0, 100, and 700 s/mm^2^ (Δ 32.9 ms, δ 6.1 ms, repetition time 6000 ms, echo time 67 ms, inversion time 116 ms, 20 slices, slice thickness 5 mm, no gap, voxel size 3.5 × 3.6 × 5.0 mm^3^, phase-encode direction right-left, number of signal averages 2, ratio b-value averages 1:1:2, SENSE factor 1.6 (left–right), scan time 2 min, 6 s). Sequences were planned so that the central slice passed through the MR markers.

After the baseline repeatability measurements, a total of 27 separate sonications of 2 to 12 mm cell diameter were made in the six lamb leg samples at a frequency of 1.2 MHz. PRFS data were obtained during sonications. The acoustic power of sonications ranged from 20 W up to the maximum allowable for each cell size. The exposure duration was fixed for each cell diameter, as shown in Table 1.

**Table 1:** Applied acoustic power and energy, estimated thermal dose volume (V_240EM_), and maximum recorded tissue temperature for 27 separate sonications using from 2 mm to 12 mm diameter cells for exposure durations ranging from 16 to 36 s in *ex-vivo* lamb legs (*n* = 6)

Cell diameter/exposure duration	Power (W)	Energy (kJ)	V_240EM_ (cm^3^)	Maximum temperature (°C)
2 mm/16 s	90	1.44	0.83	81.2
	120	1.92	2.63	67.2
	150	2.40	3.06	94.9
	190	3.04	3.57	112.4
	190	3.04	2.87	101.4
4 mm/16 s	20	0.32	0.00	42.1
	40	0.64	0.15	61.7
	60	0.96	0.16	67.2
	80	1.28	0.65	71.6
	120	1.92	0.74	77.1
	150	2.40	4.10	92.6
	160	2.56	2.28	113.6
	190	3.04	2.59	98.9
	190	3.04	4.12	113.2
8 mm/20 s	20	0.40	0.03	62.5
	40	0.80	0.39	58.8
	40	0.80	0.61	54.6
	60	1.20	1.16	69.1
	80	1.60	0.90	68.8
	120	2.40	3.36	77.1
	150	3.00	5.14	78.7
	150	3.00	4.38	81.8
12 mm/36 s	20	0.72	0.21	59.7
	40	1.44	2.72	89.9
	60	2.16	3.92	74.1
	80	2.88	5.46	81.2
	80	2.88	5.75	81.1

In each case, the highest power exposure was delivered in two different samples

After each sonication, the DWI sequence was acquired five more times at approximately 4 min intervals. The first was immediately after the mandatory thermal cooling monitoring period and the last approximately 20 min after sonication. The process was repeated for every sonication in each lamb leg during each imaging session. As the DWI sequence was a multi-slice acquisition, DWI acquired after sonications delivered towards the end of imaging sessions also provided delayed data up to 2 h after some of the earlier exposures, if they were included within the field of view.

On completion of all sonications in each lamb leg, three-dimensional gradient-echo T1-weighted images were re-acquired to assess the conspicuity of any thermal lesions that had been generated. An echo time of 2.3 ms and flip angle of 20^o^ were selected to maximise contrast between lesions and normal tissue, based on the expected T1 values of muscle tissue at 3T [22]. In addition, for maximum potential lesion conspicuity, an attempt was made to generate large thermal lesions in lamb leg number 8 by doubly sonicating two 4 mm cells at 160 W and by sonicating two 8 mm cells at 160 W and 200 W, achieved by operating the Sonalleve in *uterine-mode*, rather than *bone-mode*. After sonications, DWI and T1-weighted sequences were acquired. In addition, T1-weighted fat suppressed volume interpolated and T2-weighted images with and without fat suppression were obtained. In each case, parameters were selected to maximise image contrast between thermal lesions and un-sonicated regions, e.g. using a short echo time of 40–60 ms in the T2-weighted sequences, for an expected muscle T2 value of approximately 50 ms [22].

### Validating imaging findings with macroscopy

After imaging, each of the seven lamb legs was sliced with reference to the MR markers to determine whether any macroscopic thermal change could be visualised. Expected appearances were of regions of pale tissue against a background of darker red tissue.

### Data analysis

#### Baseline temperature measurements

For each frame of the PRFS acquisitions, the maximum temperatures recorded in the monitoring slice in the coronal, sagittal, and transverse planes were plotted as time intensity series. Mean and standard deviation (SD) values of all recorded measurements summarised the accuracy and precision of the PRFS measurement before sonication. After sonications, the regression equations for the cooling portion of these curves were used to calculate the time by which temperatures would return to baseline after exposures.

#### Imaging experiments

**Fig. 2:**
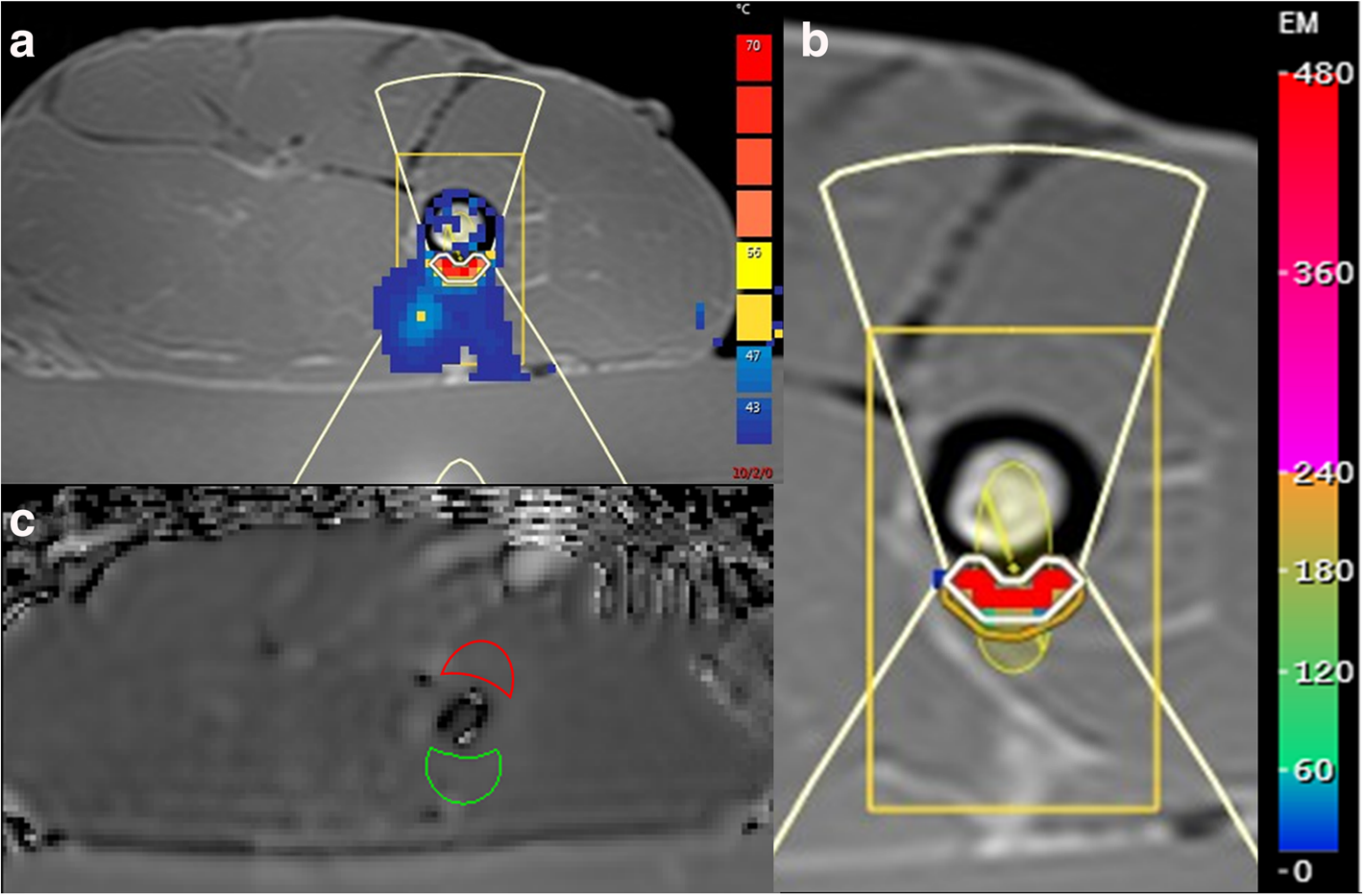
**a** T1-weighted image showing a lamb leg sample placed on a gelpad for sonication, with the heated region indicated by a colour overlay of the temperature map derived from PRFS (red pixels > ~60 °C). The sonicated cell (yellow ellipsoid) is shown magnified for greater clarity in (**b**), with the heated region shown in units of thermal dose (red pixels > ~400 equivalent minutes (EM)). The white line indicates the 240 EM at 43 °C thermal dose contour. ROI positions representing the heated region on the relevant slice of the ADC map (green outline), and a control region placed distally (red outline) are shown in (**c**)

ADC maps were calculated only for b-values of 100 and 700 s/mm^2^; b = 0 s/mm^2^ data were excluded. The DWI slice position coinciding with each planned treatment cell was identified. Regions of interest (ROIs) were then drawn on the relevant slice of the ADC maps to encompass the heated regions seen on PRFS thermometry (Fig. 2) and were subsequently copied to the equivalent regions on ADC maps generated at each time point, including the repeat baseline acquisitions. The mean ADC values for the ROIs drawn for each cell size at a range of sonication powers at each time point were plotted as time intensity series and compared. Time after sonication was calculated as the time interval between the end of sonication and the start of DWI acquisition. To determine whether any ADC changes were seen in muscle tissues outside the heated focal regions, an additional control ROI was drawn at the distal cortical surface and ADC values recorded at every time point for the two highest power exposures at each cell size.

PRFS provided spatio-temporal temperature maps. By accumulating these maps over time, the Sonalleve software yielded thermal dose data that were displayed as 240 and 30 equivalent minutes (EM) at 43 °C [3] dose contours, overlain on the anatomical imaging. The three orthogonal maximum dimensions of the 240 EM at 43 °C dose contour were measured because they are designed to represent the contour within which thermal damage will form. The product of these measurements was used as the estimate of thermal dose volume (V_240EM_). This rectangular volume was used in volume comparisons for simplicity, in preference to assuming ellipsoidal geometry and multiplying by a constant (4π/3). The maximum temperature estimated by PRFS in the target region during each sonication was also recorded.

### Statistical analysis

Statistical analysis was performed using SPSS (IBM SPSS Statistics for Windows, Version 23.0. Armonk, NY, USA) and GraphPad Prism (GraphPad Software, Version 7, San Diego, USA). Normality plots, Kolmogorov-Smirnov, and Shapiro-Wilk tests for normality were used to determine whether parametric or non-parametric tests had to be used. *p* values lower than 0.05 were considered as significant.

#### Repeatability of ADC measurements

A Bland-Altman plot was used to compare mean and percentage difference in ADC from pairs (*n* = 10) of baseline measurements of mean ADC recorded in spatially matched ROIs. Repeatability was estimated by calculating the coefficient of variation and 95% limits of agreement (LoA) for these measurements.

#### Temporal evolution of ADC change

ADC changes were classified as significant if they exceeded the 95% LoA established from the repeatability measurements. The assessment was made at each of six time intervals (< 1, 1–5, 6–10, 11–15, 16–20, and 21–50 min after sonication). Independent sample *t* tests and receiver operating characteristic (ROC) analysis were used to examine differences in ADC change 1–5 min after sonication between cases with and without significant ADC changes that persisted for more than 20 min.

#### Relationship between prescribed and measured thermal parameters

The product of acoustic power and the duration of each sonication was used to calculate the applied acoustic energy for each exposure. Acoustic energy was then compared with PRFS-estimated thermal parameters by correlating it with V_240EM_ and maximum temperature. Spearman’s correlation was used for the non-normally distributed V_240EM_ data because a normal distribution was not achieved by transforming the data.

#### Relationship between ADC change and thermal parameters

The strength of any relationship between ADC change and thermal parameters was assessed by correlating ADC change at the specified time intervals with the applied acoustic energy, V_240EM_, and maximum temperature. Independent sample *t* tests and ROC analysis were used to examine differences in thermal parameters between cases with and without significant ADC changes that persisted 20 min after sonication. Sonications were then separated into those delivered above and those below these ROC-established thermal thresholds. ADC change in each group was compared using paired *t* tests with Bonferroni correction for multiple comparisons.

## Results

### Baseline temperature measurements

**Fig. 3:**
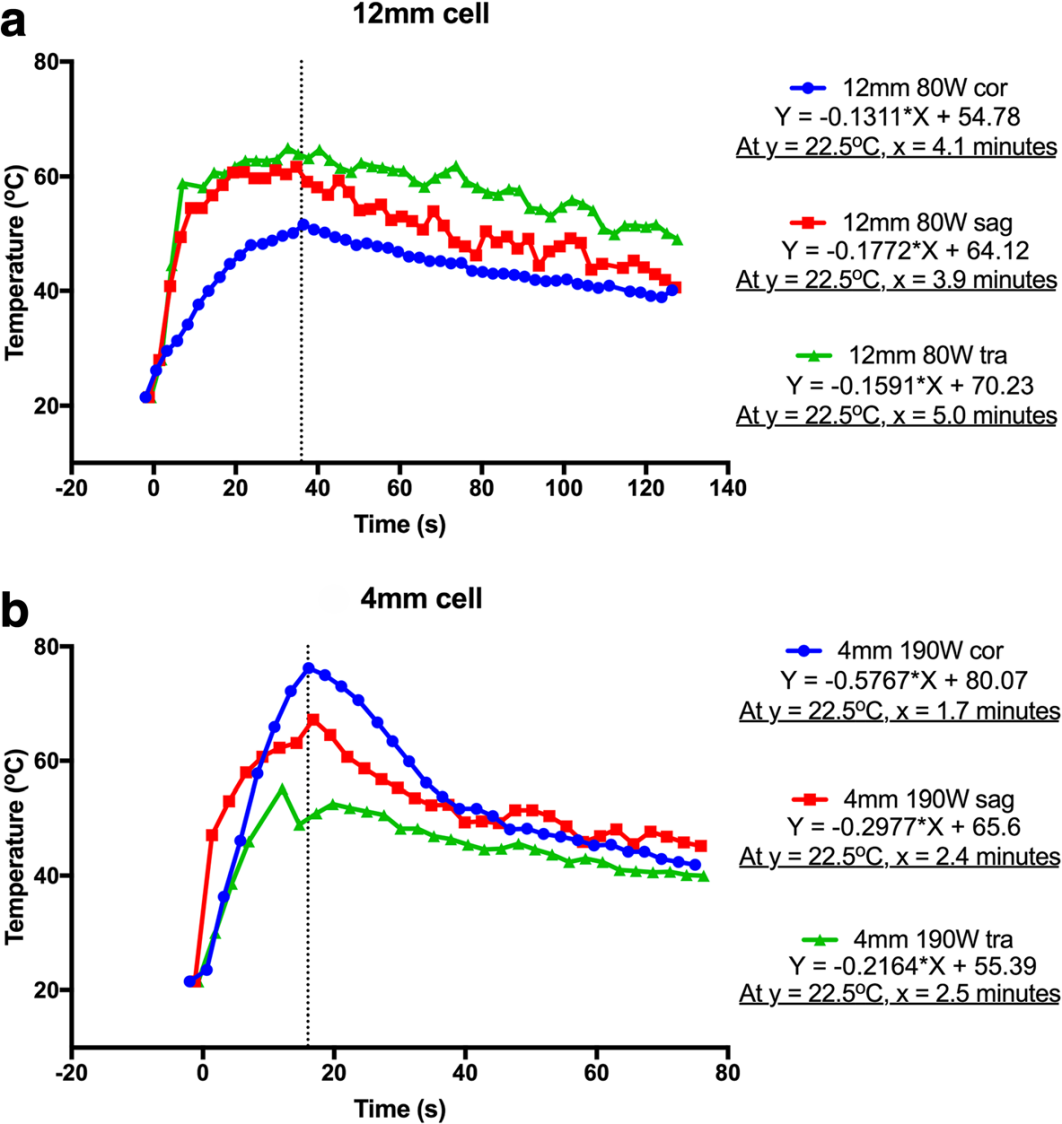
Time versus temperature curves from data acquired at the focus in the coronal (cor; blue), sagittal (sag; red), and transverse (tra; green) planes for **a** 80 W exposure of a 12 mm diameter cell over 36 s, and **b** 190 W exposure of a 4 mm diameter cell over 16 s. Solving the regression equations for the cooling portions of the curves (to the right of the black dashed lines) indicated that tissue temperature would return to baseline (22.5 °C as measured by PRFS) within 5 min for a 12 mm cell and 2.5 min for a 4 mm cell

Prior to sonications, the temperature recorded from baseline PRFS measurements (*n* = 138) in lamb leg sample number 1 was 22.5 ± 0.5 °C (mean ± SD) compared to the measured room/lamb leg temperature on that day (21.5 °C). After sonications, PRFS measurements indicated that tissue temperature would return to baseline by 5 min after an 80 W exposure of a 12 mm cell, and by 2.5 min after a 190 W exposure of a 4 mm cell (Fig. 3).

### Imaging experiments

#### Repeatability of ADC measurements

**Fig. 4:**
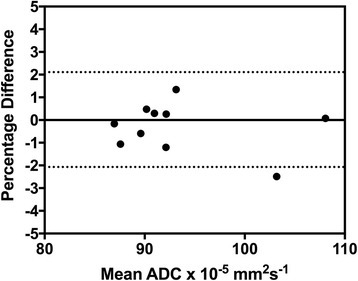
Bland-Altman plot from 10 pairs of baseline measurements of mean apparent diffusion coefficient (ADC) in ROIs copied from those later drawn in heated regions identified on PRFS after sonications. The 95% limits of agreement (from 2.1% to −2.1%) are indicated by the dashed black lines

Baseline mean un-sonicated ADC values of muscle regions directly overlying the periosteum for ROIs (*n* = 27) ranged from 83.7 to 110.0 × 10^−5^ mm^2^/s (99.5 ± 6.9 × 10^−5^ mm^2^/s, mean ± SD). The coefficient of variation for *n* = 10 repeat baseline measurements was 0.8% (95% LoA from −2.1% to 2.1%) (Fig. 4).

#### Temporal evolution of ADC change

**Fig. 5:**
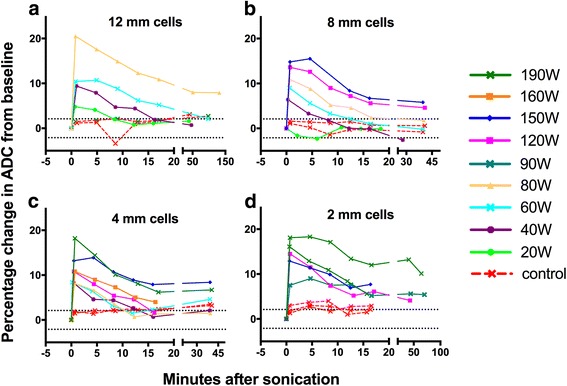
ADC time-intensity series for **a** 12 mm, **b** 8 mm, **c** 4 mm, and **d** 2 mm diameter cells. For each cell size, the magnitude of ADC change immediately after sonication was related to the acoustic power, with sustained ADC changes higher than limits of agreement (black dashed lines) only seen when the applied acoustic energy (determined by the power and duration of the exposure) exceeded approximately 1.5 kJ. Similar ADC changes were not seen in the control ROIs (red dashed lines). For image clarity of the separate time-intensity curves, the 20 W exposure data are not shown for the 4 mm cell

As expected, the periosteum was not resolved on DWI, but ADC changes were observed in the muscle tissues overlying the periosteum after sonication. ADC time-intensity series for 2, 4, 8, and 12 mm cells are shown in Fig. 5. These indicate that the maximum ADC increases occurred within the first 5 min after sonication, when temperatures would have been elevated. At a power of 80 W, there was a maximum increase in ADC of 20% with 12 mm cells (2.88 kJ), 10% with 8 mm cells (1.6 kJ), and 7% with 4 mm cells (1.28 kJ). The increase in ADC was always greater than LoA from the repeatability measurements immediately after sonication, but remained elevated more than 20 min after sonication in half of the cases (Table 2), when tissue temperatures had returned to baseline. In samples with persistent and significant ADC increases at 20 min, the percentage change in mean ADC 1–5 min after sonication was significantly greater than in samples without persistent ADC changes (11.3 ± 4.9% versus 5.8 ± 3.4%, *p* = 0.009). ROC analysis showed that a 9% increase in ADC 1–5 min after sonication separated samples with significant ADC increases that persisted 20 min after sonication from those that did not with 69% sensitivity and 89% specificity (area under the curve (AUC), 0.80). After 20 min, ADC changes were stable up to 2 h after sonication. Evaluation of the control ROIs placed at the distal cortical surfaces showed that ADC change in un-sonicated regions was comparable with the LoA in each case.

**Table 2:** Number of cases out of 27 exposures with significant ADC changes (greater than ±2.1% limits of agreement estimates, established from the pairs of baseline measurements) at each of the evaluated time intervals

Time after sonication (min)	Number of cases out of 27 with significant ADC change
< 1	26^a^
1–5	26
6–10	22
11–15	21
16–20	17
21–50	13^b^

^a^ Diffusion-weighted imaging (DWI) data were not available in one case due to a slightly prolonged mandatory cooling time

^b^ DWI data were not available in five cases because exposures were made close to the end of the imaging session

These data show that apparent diffusion coefficient (ADC) increases were always significant immediately after exposure, and that significant changes persisted more than 20 min after exposure in half of the cases

#### Relationship between prescribed and measured thermal parameters

Applied acoustic energy for the exposures (*n* = 27) ranged from 0.32 to 3.04 kJ (1.90 ± 0.94 kJ, mean ± SD). Measured V_240EM_ ranged from 0 to 5.75 cm^3^ (2.29 ± 1.84 cm^3^), and the recorded maximum tissue temperature ranged from 42.1 to 113.6 °C (79.0 ± 18.4 °C) (Table 1). There were strong and significant correlations between applied acoustic energy and V_240EM_ (ρ = 0.85, *p* < 0.001) as well as between applied acoustic energy and maximum tissue temperature (*r* = 0.80, *p* < 0.001).

#### Relationship between ADC change and thermal parameters

**Fig. 6:**
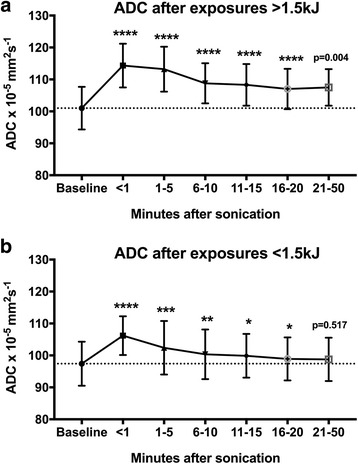
Apparent diffusion coefficient (ADC) values (mean ± SD) as a function of time after sonication for **a** delivered energies above 1.5 kJ remained significantly higher than baseline (dashed line) 21–50 min after sonication (*p* = 0.004), whereas **b** for exposures below this threshold value had almost returned to baseline values at this time point (*p* = 0.517). ****p < 0.0001, ***p < 0.001, **p < 0.01, *p < 0.05

At every specified time interval there were significant correlations between percentage ADC change and applied acoustic energy, V_240EM_, and maximum temperature (Table 3). The strength of these correlations across the six specified time points ranged from *r* = 0.55–0.85 (ADC and energy), *r* = 0.48–0.80 (ADC and V_240EM_), and *r* = 0.44–0.60 (ADC and temperature). In 16/17 cases with significant ADC increases persisting 16–20 min after sonications, exposures had been delivered at acoustic energies > 1.5 kJ (the mean ± SD acoustic energy for cases with persistent, significant ADC changes [n = 17] was 2.46 ± 0.65 kJ, compared with 0.95 ± 0.50 kJ for cases without persistent, significant changes [n = 10]; *p* < 0.001). ROC analysis showed that an applied acoustic energy of 1.52 kJ separated samples with and without significant ADC changes persisting 16–20 min after sonication with 88% sensitivity and 90% specificity (AUC, 0.96). In addition, a thermal dose volume with a V_240EM_ of 1.72 cm^3^ and a maximum temperature of 68.0 °C separated the two groups with 83% sensitivity and 90% specificity (AUC, 0.92) and 88% sensitivity and 60% specificity (AUC, 0.88), respectively. For sonications delivered with energy > 1.5 kJ, there was a significant difference between ADC at baseline and ADC more than 20 min after sonication (*p* < 0.001). At that time, the difference was not significant for sonications delivered with energy < 1.5 kJ (*p* = 0.517) (Fig. 6).

**Table 3:** Correlations between percentage change in apparent diffusion coefficient (ADC) and applied acoustic energy, thermal dose volume (V_240EM_), and maximum temperature, showing that statistical significance was achieved at every measured time point, although the strength of the correlations varied

Time after sonication (min)	Correlation between % change in mean ADC and:		
	Applied acoustic energy	V_240EM_^a^	Maximum temperature
< 1	*r* = 0.73	ρ = 0.61	*r* = 0.50
	*p* < 0.001	*p* = 0.001	*p* = 0.010
1–5	*r* = 0.85	ρ = 0.80	*r* = 0.57
	*p* < 0.001	*p* < 0.001	*p* = 0.002
6–10	r = 0.55	ρ = 0.48	r = 0.44
	*p* = 0.003	*p* = 0.011	*p* = 0.023
11–15	r = 0.77	ρ = 0.73	*r* = 0.53
	*p* < 0.001	*p* < 0.001	*p* = 0.005
16–20	r = 0.77	ρ = 0.76	r = 0.53
	*p* < 0.001	*p* < 0.001	*p* = 0.005
21–50	*r* = 0.66	ρ = 0.52	*r* = 0.60
	*p* = 0.001	*p* = 0.011	*p* = 0.003

^a^ Non-parametric Spearman’s test was used to correlate non-normally distributed V_240EM_ data

#### Validation of imaging findings with macroscopy

**Fig. 7:**
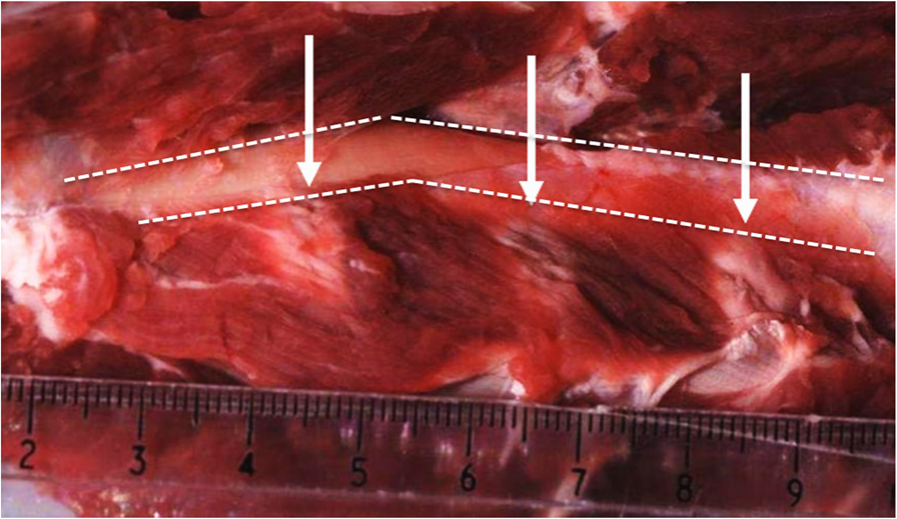
Three of four thermal lesions seen after exposures to lamb leg sample 8. Macroscopic tissue changes were seen as focal regions of pale muscle tissue (white arrows) adjacent to the bone surface whose approximate outline is indicated by the white dashed lines. In these muscle tissue lesions, post-sonication ADC values were 20% higher than the pre-sonication measurements

In lamb leg number 8, clear visible macroscopic lesions could be identified as focal regions of pale tissue (Fig. 7); these lesions were not visible on T1- or T2-weighted images. ADC increases of 18.1%, 21.9%, 20.7%, and 21.1% (mean 20.2%) were measured in these regions after the four exposures and were sustained 30 min after sonication. In lamb leg sample numbers 2 to 7, macroscopic lesions were also found after sonications at 120–190 W (from 1.9 to 3.0 kJ), for which ADC initially increased by 10–15%. However, difficulties in slicing the lamb legs prevented accurate identification of the lower threshold at which permanent tissue damage occurred.

## Discussion

Our baseline temperature measurements supported the already reported accuracy of PRFS thermometry to approximately 1 °C [23] and indicated that tissues cooled to baseline temperatures within 5 min after sonication, even when using the largest cell size and highest exposure parameters. The ADC measurement at baseline was found to be highly repeatable, although this may have been influenced by the lack of active perfusion in the lamb leg samples.

After sonication, focal ADC increases on DWI were observed in the target tissues, which were maximal in the first 5 min after sonication when tissue temperatures would have been elevated. This was unsurprising, as the temperature dependence of ADC is well known, with a 2.4% ADC change expected per 1^o^ C temperature change [21]. However, in half of cases ADC changes were still present 20 min after exposure, by which time temperatures would have returned to baseline. Therefore, these ADC changes were probably related to early thermal damage to the periosteum and adjacent muscle tissues overlying the bone. In fact, the magnitude of ADC change seen 1–5 min after sonication was indicative of sustained ADC change at 20 min, with reasonable sensitivity and specificity (AUC, 80%). Delayed measurements also showed that there was no further change in ADC between 20 min and 2 h after exposure.

At every measured time point, ADC changes were significantly correlated with applied acoustic energy and with thermal dose volume and the maximum temperature recorded. Some pre-clinical studies have also compared DWI changes with thermal dose estimates during prostate treatments [24, 25] but the relationship between ADC and thermal dose has not been previously described in bony regions. Moreover, the clear differences in thermal parameters between cases with and without significant ADC changes at 20 min mean that intra-procedural estimates of thermal dose volume can be directly used to predict the final extent of tissue damage.

Clear focal ex-vivo muscle tissue change was confirmed macroscopically when ADC increases of 20% were measured. The ADC increase is consistent with a breakdown of cell membranes, expected after thermal damage [26], although there was no concomitant change in T1W or T2W images. Unfortunately, contrast-enhanced imaging could not be obtained in these ex-vivo samples to confirm non-perfusion or tissue necrosis. In-vivo, contrast enhancement is the most robust way of demonstrating tissue ablation at the end of the procedure [5], but intra-procedural contrast enhanced imaging is not feasible [7].

*Ex-vivo* lamb legs were used in this study because they represented a reasonable model without resorting to in-vivo animal models and allowed for macroscopic inspection of sliced tissues after exposure. The model was not ideal because there was no active perfusion, and samples were not at a physiological temperature. In vivo, it is likely that perfusion would contribute substantially to tissue cooling. The dissipation of heat may mean that the relationship between thermal dose, ADC, and tissue damage is influenced by local blood supply [27]. We are therefore extending the current work by evaluating the repeatability, temporal evolution, and permanence of ADC changes during MR-guided HIFU treatments for palliating pain from bone metastases in patients recruited to a clinical study [28] at our institution.

In patients, the presence of sustained DWI changes in muscle regions overlying the periosteum after sonication would imply that thermal neurolysis has been achieved at the periosteum. However, these changes also represent damage to the overlying muscle which could induce unwanted symptoms after treatments, such as stiffness or weakness [29]. An early warning of the potential for muscle damage could allow operators to modify treatments to ensure patient safety or prompt proactive, early referral to physiotherapy services to manage and minimise symptoms.

This current ex-vivo study serves to inform our clinical in-vivo findings by providing preliminary data on the relationship between thermal dose, temperature, diffusion-weighted change, and macroscopic tissue damage.

Limitations of this study relate to inaccuracies in estimating the thermal and imaging parameters. Applied acoustic energy takes no account of the depth-dependent loss due to attenuation in overlying tissues [30]. In this study, uniformity of samples meant there was little variation in these factors, but in clinical applications this is likely to require greater attention. The estimate of V_240EM_ did not take into account the non-rectangular shape of thermal dose volumes, implying an overestimate. However, other authors have also used the measured dimensions of V_240EM_ at 43 °C thermal dose contour as a basis for estimation of thermal dose size [31]. More accurate methods for measuring dose volume could have been employed by exporting the PRFS data off-line. For example, extraction of temperature versus time data for each individual pixel can be used to compute a thermal dose map from which it may be possible to derive a more precise thermal dose volume. Although potentially more accurate, these more complex analyses would not be available within a clinically useful time frame during treatments. Ideally, vendor-generated thermal dose volumes could be supplied as a summary parameter after each sonication. Another limitation was that the maximum temperature recorded for each sonication arose from the maximum temperature seen in any single voxel in the target region and appeared unlikely to be accurate in some cases (e.g. where recorded as > 100 °C). This bias was accepted in our analyses, i.e. the data were not censored to a maximum of 100 °C. Even when accurate within the single voxel, the value may not have been representative for the whole region. Finally, the DWI sequence was designed for rapid acquisition using an EPI-based technique and resulted in image distortions [32]. These may have led to spatial mis-registration between heated regions on PRFS and the ADC maps. Further optimisation of the DWI sequence or the consideration of fast spin-echo-based DWI sequences would reduce distortions [33].

In conclusion, our preliminary results highlight the potential of DWI for detecting intra-procedural changes in muscle regions overlying the periosteum during MR-guided HIFU bone treatments. ADC measurements in these regions were highly repeatable. The magnitude of ADC change 1–5 min after sonication was indicative of sustained ADC changes at 20 min, after which changes remained stable. These ADC changes correlated with both applied and measured thermal parameters. ADC changes > 9% at 1–5 min achieved sustained macroscopic change; changes > 20% caused increasing focal damage to overlying muscle. Intra-procedural DWI acquisitions therefore could be exploited during clinical MR-guided HIFU bone treatments without extending treatment time and may be informative about both safety and efficacy of treatments.

## Abbreviations

ADC: Apparent diffusion coefficient
AUC: Area under the curve
DWI: Diffusion-weighted imaging
EM: Equivalent minutes
EPI: Echo planar imaging
HIFU: High-intensity focused ultrasound
LoA: Limits of agreement
MR: Magnetic resonance
PRFS: Proton resonance frequency shift
ROC: Receiver operating characteristic
ROI: Region of interest
SD: Standard deviation
